# Quality of outcome reporting in phase II studies in pulmonary tuberculosis

**DOI:** 10.1186/s13063-015-1050-1

**Published:** 2015-11-14

**Authors:** Laura Jayne Bonnett, Geraint Rhys Davies

**Affiliations:** Department of Biostatistics & Department of Clinical Infection, Microbiology & Immunology, University of Liverpool, Waterhouse Building, Block F, 1-5 Brownlow Street, Liverpool, L69 3GL UK; Department of Clinical Infection, Microbiology, & Immunology, University of Liverpool, Ronald Ross Building, 8 West Derby Street, Liverpool, L69 7BE UK

**Keywords:** Tuberculosis, systematic review, outcomes

## Abstract

Tuberculosis (TB) remains a major killer amongst the infectious diseases. Current treatment involves a four-drug regimen for at least 6 months. New drugs and regimens are required to shorten treatment duration, reduce toxicity and combat drug resistance, but the optimal methodology to define the critical path for novel regimens is not well defined. We undertook a systematic review to summarise outcomes reported in Phase II trials of patients with newly diagnosed pulmonary TB to assess the need for a core outcome set. A systematic search of databases (PubMed, MEDLINE, EMBASE and LILACs) was conducted on 1 May 2015 to retrieve relevant peer-reviewed articles. Reference lists of included studies were also searched. This systematic review considered all reported outcomes. Risk of bias was considered via sequence generation, allocation concealment, blinding, reasons for exclusions, and selective reporting. Of 55 included studies, 20 were Phase IIB studies based on culture conversion, 32 were Phase IIA studies based on quantitative bacteriology, and three considered alternative outcomes. Large variation in reported outcomes and trial characteristics was observed across the included studies. Bacteriological results were as often expressed in terms of positivity as negativity, with varying definitions of culture conversion. Variation in reporting was particularly marked for Phase IIA studies, where multiple time intervals were typically selected for analysis and sometimes resulted in differing interpretations of the efficacy of drugs or regimens. Within both Phase IIA and IIB studies, there was variation in the time points at which the study participants were sampled, as well as in the bacteriological media and methods used. For successful future meta-analysis of early-phase studies, the findings of this review suggest that development of a core outcome set would be desirable. This would enable trial results to be more easily compared and combined, potentially leading to more effective development of new treatment strategies for patients with TB. Pending development of, and agreement on, such a core outcome set, we suggest some interim recommendations for reporting of future phase II studies of pulmonary tuberculosis.

## Background

Treatment of tuberculosis (TB) has evolved very slowly in the 40 years since current standard first-line regimens were first developed. Combinations of up to four drugs are needed for 6 months or more in order to ensure a relapse-free cure for most patients. Screening novel treatment regimens in Phase II trials is challenging because early indications of treatment efficacy rely on bacteriological biomarkers whose relation with long-term outcomes is unclear. Furthermore, this information can be obtained and expressed in a number of different ways, leading to a lack of consistency and comparability in the outcomes reported across clinical trials. These uncertainties are a critical risk to decision-making about which regimens should progress to Phase III trials, which are large, prolonged and expensive.

Possible bacteriological outcomes in TB are diverse. *Mycobacterium tuberculosis* may be grown on solid or liquid media of different types using different processing and decontamination methods. The laboratory results may be expressed as culture positivity at single time-points, hazard of culture conversion over multiple time-points, changes in or rate of elimination of counts of colonies on solid media, and time-to-positivity in automated liquid culture systems. Furthermore, the method of analysis may vary accordingly with the type of endpoint from simple analysis of proportions to linear or non-linear models of colony counts or liquid cultures to time-to-event analysis of culture conversion. Typically, proof-of-concept Phase IIA studies utilise quantitative bacteriology to study monotherapy over study periods no longer than 14 days (‘early bactericidal activity’ or EBA), whereas Phase IIB studies of drug combinations utilise a wider variety of methods over a longer time period, usually the first 56 days of treatment with a particular focus on culture conversion at 2 months, which has historically correlated well with long-term outcomes [1, 2]. Recently however Phase IIA studies of combination regimens have become more common.

This diversity is potentially problematic in a therapeutic area in which there is relatively limited capability to conduct large or multiple trials of the same regimen and where the relationship of short to long term endpoints is incompletely understood. Without comparability of outcomes across trials considering new regimens, it may be challenging to synthesize evidence effectively and draw the methodological conclusions necessary to improve the conduct of future trials. These issues have become pressing in light of recent prominent disappointments in Phase III studies and with the emerging need in the field to evaluate novel combinations of drugs efficiently [3]. With this in mind, we systematically reviewed the literature of Phase II studies in pulmonary TB to determine how outcomes are currently defined, how commonly they are used in published studies to date, and how they are employed in the current drug development pathway.

## Review

### Methods

Randomised controlled trials, or quasi-randomised trials, were included in our systematic review of the available literature. Included studies had to include patients with smear- and culture-positive pulmonary tuberculosis that were being treated for the first time, or had known isoniazid mono-resistant organisms on susceptibility testing. Only trials including regimens containing any combination of historic (rifampicin (R), isoniazid (H), pyrazinamide (Z), ethambutol (E), thiacetazone (T), para-aminosalicylic acid (P), and streptomycin (S)), or novel drugs used or proposed for use in first-line treatment regimens (rifabutin (Rb), rifapentine (Rp), levofloxacin (L), ofloxacin (O), gatifloxacin (G), moxifloxacin (M), bedaquiline (J), and PA-824 (Pa)) were considered.

A systematic search of databases (PubMed, MEDLINE, EMBASE and LILACs) was conducted on 1 May 2015 to retrieve relevant peer-reviewed articles. The employed search strategy drew upon common phrases and terms used in the literature. Keywords (appropriately truncated to allow a wide search) were combined with medical subject headings (MeSH) to comprehensively search four databases. The PubMed inclusive search strategy was as follows, with relevant modifications made as necessary for the other databases:

1. Search (tuberculosis) AND clinical trials
2. Search ((((((((((((((rifampicin) OR isoniazid) OR pyrazinamide) OR ethambutol) OR thiacetazone) OR pyrazinamide) OR streptomycin) OR rifabutin) OR rifapentine) OR levofloxacin) OR ofloxacin) OR gatifloxacin) OR moxifloxacin) OR bedaquiline) OR PA-824
3. Search (#1) AND #2

No language restrictions were imposed. The search strategy was supplemented by hand searching reference lists of included studies and relevant reviews. One author (GD) reviewed the title and available abstract for all identified citations to determine relevance. Another author (LB) repeated this process on 1 May 2015 to check for additional studies. Following the initial review, both authors (LB and GD) independently reviewed full-text publications to make a final selection of included Phase II studies evaluating either monotherapy or combination regimens. A structured form was used to record relevant information and ensure uniformity of evaluation for each study. Extracted data included study characteristics including country of study, sample size, treatments (including dosages and regimens), and all reported outcomes. Risk of bias was considered via sequence generation, allocation concealment, blinding, reasons for exclusions, and selective reporting.

### Results

**Fig. 1:**
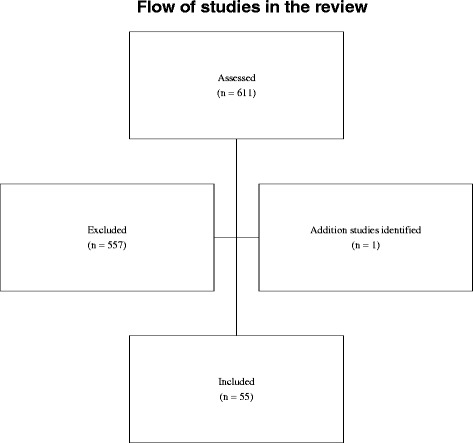
Flow of studies in the review

The flow of studies through the review is shown in Fig. 1. The main reasons for exclusion were failure to meet the inclusion criteria, and study design other than randomised controlled trial. In total, 55 relevant studies were identified and included.

**Fig. 2:**
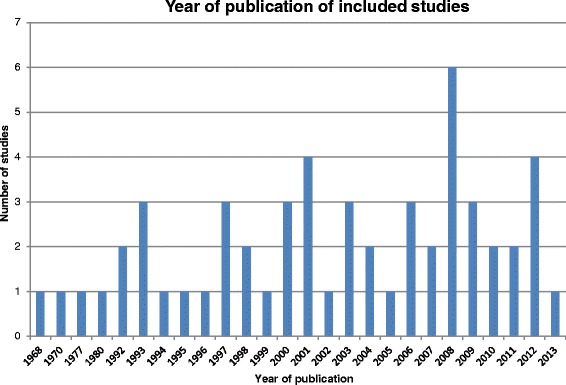
Year of publication of included studies

A bar chart summarising the year of publication of the included studies can be seen in Fig. 2. In 1996, CONSORT guidelines were first published for transparent reporting of clinical trials [4]. The majority (79 %) of studies included in our review were published after 1996, and consequently should conform to the CONSORT guidelines and present thorough information on items such as trial design, intervention, participants, and outcomes, which must be completely defined [4].

Despite the CONSORT guidelines, a core outcome set for TB that defines a minimum set of clearly defined outcomes to be reported in each future study [5] has not yet been developed. Consequently, there is wide variation in the definition and type of outcomes reported. Most studies included in our review reported more than one outcome, and the distinction between primary and secondary outcomes was often unclear. Therefore all outcomes included in each study have been considered and are summarised in Table 1.

**Table 1:** Reported outcomes

Outcome	Definition	No. studies reporting outcome
EBA	Fall or rate of change in log_10_ CFU/ml sputum	36
CFU count	Mean concentration of viable bacilli	11
AFB	Acid-fast bacilli	3
Culture negativity	Proportion, or time to, culture negativity	8
Culture positivity	Proportion, or time to, culture positivity	10
Smear/culture conversion	Proportion converting, or time to conversion	9
Sterilisation	Proportion or time to sterilisation	2
Other culture outcome	For example, persistence, relapse, time to detection, culture status	4
Adverse events		5
Gaffky code		1
Contamination	Of cultures	1
Mortality		1
Symptom disappearance	Proportion with, or time to symptom disappearance	1

The methods and purposes of Phase IIA and IIB studies in tuberculosis differ, and we have considered these study types separately in the quantitative review that follows. Of the 55 included studies, 32 were Phase IIA studies, 20 were Phase IIB studies, and three were designed to consider alternative outcomes such as contamination [6], and Gaffky code - a numerical rating for the classification of tuberculosis according to the number of tubercle bacilli in the sputum [7]. One study considered both Phase IIA and IIB outcomes [8].

In addition to the differing types of phase II studies, different culture media were considered across trials. For example, 34 studies (26 Phase IIA studies, five Phase IIB studies, one study considering EBA and culture together [8], and two that considered alternative outcomes [6, 9]) reported results obtained using solid media such as Lowenstein-Jensen and Middlebrook 7H10. In 12 cases (eight Phase IIB studies and four Phase IIA studies), the laboratory methodology described the use of both solid and liquid media, such as the BACTEC or MGIT system, and a single set of results combined over the multiple media were presented. Three studies (all Phase IIB studies [10–12]) described culture on both liquid and solid media but presented disaggregated results per medium. Several studies did not describe the medium used - one Phase IIA study [13], four Phase IIB studies [14–17], and one study looking at an alternative outcome [7]. Three of these studies were published pre-CONSORT [7, 14, 15]. One study was a conference abstract [13] where space constraints meant methodology could not be reported, and two were published in Russian language journals, which appeared not to adopt the CONSORT reporting guidelines [16, 17].

A range of analytic approaches to these varied data were considered, with multiple and wide-ranging methods being reported in most publications. Some authors opted to analyse their data using regression models such as logistic [12, 18] or linear [10, 19–21] analysis. More commonly, authors considered t-tests [6, 22, 23], ANOVA methodology [24–26], and chi-squared tests [11, 18, 27] for normally distributed data, or Kruskal-Wallis [28, 29], Mann-Whitney U [30, 31], or equivalent tests when the data was not normally distributed. Infrequently, time-to-event analysis methodology was used [12, 28, 32], as well as correlation methodology, including the Wilcoxon signed-rank test [8, 30, 31]. None of the studies were adjusted for multiple comparisons.

Regarding risk of bias, 24 (44 %) studies did not report the method of sequence generation. All but four studies (83 %) used random allocation (with stratification in some cases), rather than consecutive allocation. Only four studies (7 %) mentioned allocation concealment, mainly via opaque envelopes. Six studies (11 %) were of a double-blind design, and another six were single-blind studies. Twenty-eight studies (51 %) provided reasons for exclusions, or numbers lost to follow-up. Seventeen (31 %) studies were published pre-CONSORT when selecting reporting was not considered as a possible source of bias. In all studies published post-CONSORT, the risk of bias is unclear, as there is insufficient information to determine whether the published reports include all expected outcomes, including those that were pre-specified.

#### Phase IIA studies

**Fig. 3:**
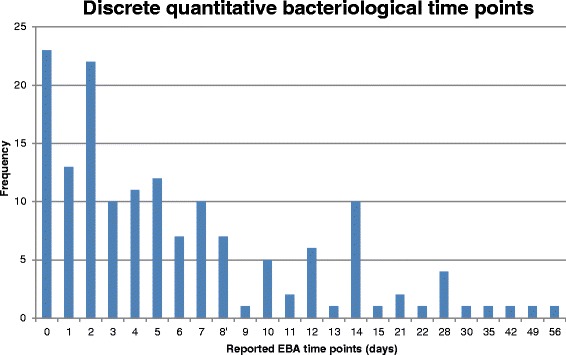
Reported time points in Phase IIA studies - discrete quantitative bacteriological time points

**Fig. 4:**
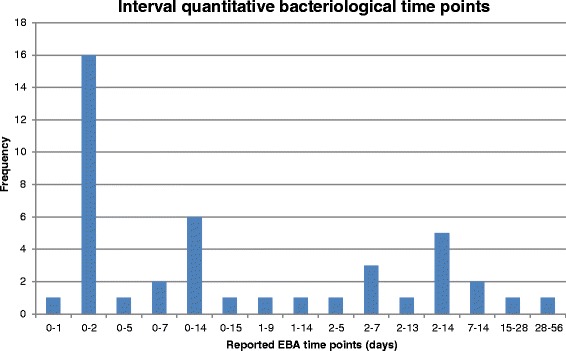
Reported time points in Phase IIA studies - interval quantitative bacteriological time points

More than half (56 %) of the included studies were designed to assess EBA, although authors did not always precisely define this term and explicit definitions differed between studies. In most cases, EBA was defined as the fall, or mean rate of change, in log_10_ colony-forming units (CFU) per ml sputum over various time periods or between two time-points. Some authors did not define their outcome as EBA but used methods that conformed to this approach, for example, decrease in sputum bacillary load of *Mycobacterium TB (M. TB)* from pre-treatment to day 15 of study drug treatment [33], or mean rate of decline of CFU [20], or decrease in viable count [34]. In one case, EBA was reported over 8 weeks [8]. Figure 3 and Fig. 4 summarise the reported time points in Phase IIA studies, showing that for the majority of studies included in this review, endpoints were focused only on the first week of treatment.

EBA studies showed a range of durations from 2 to 90 days. Seven studies lasted 2 days, three lasted 7 days, and seven lasted 14 days. Other frequent durations were 5 days (six studies), 28 days (three studies), and 15 days (two studies). Infrequently chosen durations were 8, 9, 30, 56 and 90 days, each of which was used for individual studies only. EBA results were most frequently reported in a table with differing time intervals from zero to 14 days. Measures included EBA 2 to 14 days [35], 2 to 5 days [24], and 7 to 14 days [36], along with the more common 0 to 2 days, 2 to 7 days and 0 to 7 days [37]. In other studies, EBA was reported in a figure and was therefore presented at a number of time points, for example, once daily from days 0 to 5 [38] or, once daily on days 0, 1, 2, 3, 4, 6, 8, 10, 12 and 14 [35].

Some authors referred to EBA specifically as the change in log_10_ CFU/ml sputum during the first 2 days of treatment and referred to ‘extended EBA’ as the decline in bacilli during the last 5 days of study drug administration (for example, days 2 to 7) [26, 29]. Several studies, instead of reporting fall or change in log_10_ CFU/ml, defined as EBA above, reported mean concentration of viable bacilli at a fixed time point, or mean viable count (log_10_ CFU/ml) [39]. CFU count was always presented in table form, and there was better agreement among authors about the definition of this outcome. However, in one case [40] the CFU counts were standardised and in another the rate of fall of CFU counts was reported (referred to as the ‘kill index’) [41]. The time interval over which CFU counts were presented ranged from 2 days to 56 days in one case [6].

#### Phase IIB studies

Within studies designed to consider 2-month outcomes, the most frequently reported outcome (30 %) related to culture positivity. This was measured in many ways including time of last positive sputum culture or smear [9], and time to stable culture conversion. This was defined as the number of days from study treatment initiation to the time of sputum collection yielding the first negative culture that was followed by at least one subsequent negative culture and no subsequent positive culture [42]. In one study, positivity was expressed as the percentage of cultures positive at fixed time points such as at 28 days [39].

**Fig. 5:**
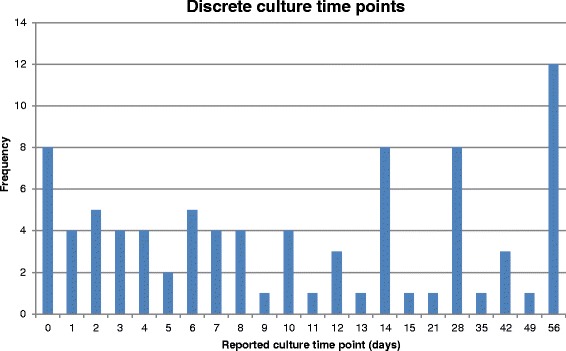
Reported time points in Phase IIB studies - discrete time points

Regarding culture negativity, whilst most (24 %) authors opted to present the proportion of negative cultures at a time point (usually 2 months), some used time to [28], or speed of [12], culture conversion. Negativity was more simply expressed as either a binary outcome at a fixed time point [27], or the proportion of patients whose culture had converted at a fixed time point [10, 42]. The definition of time to culture conversion varied between studies. One study defined time to culture conversion as the time from the start of treatment to the first of two consecutive culture negative sputum samples on non-consecutive days that were not followed by a positive sputum sample [10]. Another defined the outcome as the time point after which all sputum cultures were negative [12]. All relevant studies presented results at 2 months, but also additional time points where culture status was considered (weekly, or biweekly from zero to 8 weeks [18, 27]). Figure 5 summarises the reported time points in Phase IIB studies. Infrequently, studies reported culture conversion over a range of days, for example, 0 to 2 days.

Notably, few studies clearly reported numbers of culture samples missing due to non-attendance, sample contamination, or lack of sputum production at each time point.

#### Trial characteristics

In addition to the diversity of the outcomes reported, and the variation in their definitions, it should be noted that there was also variation across trial characteristics. As mentioned above, different time points were used for the reporting of outcomes. This adds to the complication when attempting to compare the results from multiple studies to synthesize evidence to support treatment regimens. Finally, in most cases an estimate of variability, such as a standard deviation or 95 % confidence interval was provided together with the point estimate. Some studies, however, only presented a point estimate (for example, [43]). These issues all make combining evidence from multiple studies via meta-analysis challenging.

## Conclusions

The results of this systematic review show that there is large variation in the outcomes and trial characteristics reported across phase II studies in pulmonary TB and in the statistical methods used to analyse them. Within both Phase IIA and IIB studies, there was variation in the time points at which study participants were sampled, as well as in the media used both in terms of the use of solid versus liquid media, and in the specific medium used, for example, Middlebrook 7H10 versus 7H11. Variation in reporting was particularly marked for Phase IIA studies, where multiple, apparently arbitrary, time intervals were typically selected for analysis, sometimes resulting in differing results and interpretations of the efficacy of drugs or regimens. Recently, alternative approaches have been proposed based on statistical modelling, but there is no general consensus on which approach should have priority. Phase IIB studies usually collect bacteriological samples on a weekly or bi-weekly basis. Bacteriological results were as often expressed in terms of positivity as negativity, with varying definitions of culture conversion. Though 2-month culture conversion is often used as the primary endpoint in Phase IIB studies, a number of different approaches have been used to analyse data at prior time points, singly or together. In general, logistic regression analyses of culture conversion were more common than time-to-event approaches.

In recent years, automated liquid culture systems for mycobacteria have become more widely distributed and are increasingly used in clinical trials. Culture positivity tends to persist for longer in such systems than on traditional solid culture because of their inherently greater sensitivity. While there are currently limited available data to clearly define comparability across these different methods, it seems likely that these technical differences could contribute considerable additional variability to any of the endpoints discussed here [12, 18, 27, 42]. It may therefore be important to be able to distinguish between or disaggregate data derived from these different laboratory methods.

Recently, efforts have been made to harmonize data collection standards in tuberculosis trials [44] and to standardise laboratory standard operating procedures to ensure comparability of individual patient datasets. However, if successful meta-analysis of multiple early-phase studies is to be carried out in the future, this review makes clear that development of a core outcome set would also be desirable. This would provide a minimum set of clearly defined outcomes to be reported in each study [5]. The core outcome set would not be exhaustive and trialists would have the opportunity to supplement it with any other outcomes of interest to them in any given trial. However, agreement on a meaningful core outcome set would make it easier for the results of trials to be compared, contrasted and combined in systematic reviews, ultimately facilitating understanding and accelerating improvement of treatment strategies for patients with TB.

### Recommendations

Pending development of, and agreement on, such a core outcome set, our review suggests some interim recommendations for reporting of future phase II studies of pulmonary tuberculosis.

#### Sampling scheme

We suggest that the sampling scheme should be clearly described, including information about the number and type of samples taken, in particular whether the samples were single or replicated, and the timing of sample collection. The proportion of samples missing due to contamination, failure to obtain or to produce a sample should also be reported. Whatever analysis is performed, summary results of bacteriological data at each time-point should also be reported, if necessary in a supplement to the study report.

#### Microbiological methods

Additionally, microbiological methods should be clearly described, particularly the use of solid and liquid media and the specific type or system used. It should always be possible to disaggregate results for solid and liquid media in study reports. Studies should also report percentages of cultures negative at the time points of interest and use a consistent, explicit definition of culture conversion. We recommend that this should be defined as the time-point at which the first negative culture in a series of negative cultures extending to the end of the sampling period occurs. The major advantage of prioritising culture conversion as the endpoint of interest is that it is a defined event rather than a continuing state of positivity and better suited to time-to-event analysis, which would facilitate comparison of results across studies with different sampling designs.

#### Measures of variability

Where quantitative bacteriological data are reported, measures of variability of the measurements, preferably the standard deviation, should always be reported. With the exception of 2-month culture positivity, which has support as a surrogate endpoint from meta-analyses and regulators [1], serial bacteriological measurements should be analysed using statistical approaches that provide estimates of effect independent of the particular time-points selected for the sampling scheme. This is particularly important as culture conversion rates at 2 months rise with improving efficacy of regimens, reaching near-universal culture conversion at this time point. Statistical modelling approaches to quantitative bacteriology and time-to-event methods are suitable for this purpose and would facilitate combination of data from different studies across a range of regimens with possibly widely varying efficacies.

#### Individual participant data

Finally, due to the diversity of possible analytical approaches and the standard of reporting observed, routine availability of individual patient datasets for the purposes of meta-analysis is desirable where study reports and supplementary information do not contain the data necessary to define relevant endpoints to similar definitions across studies.

Our review suggests that simple measures could greatly improve the quality of reporting of phase II outcomes but also points to the need to develop a core outcome set for early phase trials in TB which could secure more widespread agreement on outcomes amongst trialists and regulators. Such an approach, alongside other harmonisation initiatives in laboratory methods and data recording, could greatly improve the informativeness of systematic reviews in this area, enabling more confident prioritisation of regimens for evaluation in phase III trials on the basis of phase II results.

## Abbreviations

AFB: acid-fast bacilli
CFU: colony-forming units
E: ethambutol
EBA: early bactericidal activity
G: gatifloxacin
H: isoniazid
J: bedaquiline
L: levofloxacin
M: moxifloxacin
*M.TB*: *Mycobacterium tuberculosis*
O: ofloxacin
P: para-aminosalicylic acid
Pa: PA-824
R: rifampicin
Rb: rifabutin
Rp: rifapentine
S: streptomycin
T: thiacetazone
TB: tuberculosis
Z: pyrazinamide
